# Adsorption of plasma proteins and fibronectin on poly(hydroxylethyl methacrylate) brushes of different thickness and their relationship with adhesion and migration of vascular smooth muscle cells

**DOI:** 10.1093/rb/rbu008

**Published:** 2014-10-20

**Authors:** Jun Deng, Tanchen Ren, Jiyu Zhu, Zhengwei Mao, Changyou Gao

**Affiliations:** MOE Key Laboratory of Macromolecular Synthesis and Functionalization, Department of Polymer Science and Engineering, Zhejiang University, Hangzhou 310027, China

**Keywords:** poly(hydroxylethyl methacrylate), smooth muscle cells, protein adsorption, migration

## Abstract

The surface-grafted poly(hydroxylethyl methacrylate) (PHEMA) molecules were demonstrated to show a brush state regardless of their molecular length (molecular weight). Adsorption of proteins from 10% fetal bovine serum (FBS), fibronectin (Fn) and bovine serum albumin (BSA) was quantified by ellipsometry, revealing that the amounts of FBS and Fn decreased monotonously along with the increase of PHEMA thickness, whereas not detectable for BSA when the PHEMA thickness was larger than 6 nm. Radio immunoassay found that the adsorption of Fn from 10% FBS had no significant difference regardless of the PHEMA thickness. However, ELISA results showed that the Arg-Gly-Asp (RGD) activity of adsorbed Fn decreased with the increase of PHEMA thickness. By comparison of cellular behaviors of vascular smooth muscle cells (VSMCs) being cultured *in vitro* in the normal serum-containing medium and the Fn-depleted serum-containing medium, the significant role of Fn on modulating the adhesion and migration of VSMCs was verified. Taking account all the results, the Fn adsorption model and its role on linking the biomaterials surface to the VSMCs behaviors are proposed.

## Introduction

The cell migration plays a critical role in a variety of pathological and physiological processes, such as embryonic development, cancer metastasis, blood vessel formation and remolding, tissue regeneration, immune surveillance and inflammation [1, 2]. Many efforts have been paid to study the cell migration on the gradient biomaterials. So far the surfaces with chemical and physical gradients have been prepared and their influences on the cell migration have been investigated. Immobilization of various biomolecules such as extracellular matrix proteins (fibronectin (Fn) [3], laminin [4] and collagen [5, 6]), and growth factors (epidermal growth factor (EGF) [7], basic fibroblast growth factor (bFGF) [8] and vascular endothelial growth factor (VEGF) [9]), and RGD on gradient biomaterials can guide cell migration directionally as well.

The physical signal also plays an important role in modulating the cell migration. For example, the motility of fibroblasts can be governed purely by substrate rigidity (durotaxis), and can directionally move from the soft region toward the rigid side of the substrate [10, 11]. Recently, Han *et al.* [12] prepared a gradient multilayer with a similar surface chemistry (poly(styrene sulfonate sodium salt) PSS domination) but gradually changed swelling ratio by the treatment of different NaCl concentrations. It was found that the vascular smooth muscle cells (VSMCs) migrated directionally to the low hydration side at an appropriate cell-seeding density (1.5 × 10^4^/cm^2^) under the assistance of cell–cell interactions. On the other hand, the chemical environment used for specific tissue regeneration is also very influential. Recently, Wu *et al.* [13] reported that cell migration and adhesion are influenced by the density of poly(ethylene glycol) (PEG): the cells migrated faster on the moderate density of PEG-grafted surface whereas slower on the high or low density of PEG-grafted surfaces.

2-Hydroxyethyl methacrylate (HEMA) is a neutral and hydroxyl-rich monomer, which has a wide range of applications in soft contact lenses, water-miscible embedding medium, thermally cross-linkable paints, reactive adhesives and coatings, and radiation curing [14–16]. Similar to other hydrophilic polymers, poly(hydroxylethyl methacrylate) (PHEMA) exhibits excellent ability to resist protein adsorption from different single-protein solutions. Many works [17, 18] have reported that PHEMA can effectively resist nonspecific bacterial adhesion and protein adsorption from single proteins. The PHEMA brush was reported to be very stable, without decomposition or decrement in film thickness in phosphate buffered saline (PBS) for 20 days [19]. Excellent antifouling ability and high structural stability make PHEMA the promising biomaterials for biomedical applications.

The thickness gradient of PHEMA brushes with controllable slopes (8–32 nm/mm) was fabricated by surface-initiated atomic transfer radical polymerization (ATRP) and a continuous injection method. The VSMCs cells number and spreading area were all decreased along with the increase of the thickness of the PHEMA brushes on the gradient surfaces [20]. The VSMCs exhibited preferential orientation and enhanced directional migration behaviors on the gradient surface toward the direction of reduced PHEMA thickness in a gradient slope and polymer thickness-dependent manner. However, the underlying mechanism is still unclear.

When the biomaterials contact with the body fluids, those first arrive to the surface are ions and small molecules, followed with the proteins, and finally the cells. So the protein adsorption behavior on biomaterials can influence the following cell behaviors. Jan Genzer *et al.* reported that the amount of Fn adsorbed on the PHEMA-grafted substrate is correlated with the coverage of PHEMA on the substrate, and the amount decreases with increasing PHEMA coverage. The cell density and spreading on the substrate increase with the increasing Fn coverage [21]. However, it is much more complex for protein adsorption on biomaterials in serum because there are hundreds types of proteins, leading to competitive adsorption. The present study explores the relationship between serum protein adsorption and cell behaviors (adhesion and migration) on different thickness of PHEMA brushes. To the best of our knowledge, no study has been reported to correlate the serum protein adsorption from 10% FBS on PHEMA-grafted surface with a brush state to the cell adhesion and migration.

## Materials and Methods

### Materials

Bovine serum albumin (BSA), ascorbic acid (Vc), 3-triethoxysilylpropylamine (APTES), α-bromoisobutyryl bromide (BIBB, 97%), *N*,*N*,*N*′,*N*′,*N*″-pentamethyldiethylenetriamine (99%) and HEMA (97%) were purchased from Sigma–Aldrich (USA). HEMA was passed through base alumina column to eliminate hydroquinone methylether (inhibitor) before use. Dichloromethane (CH_2_Cl_2_), toluene and triethylamine (Et_3_N) were purchased from Sinopharm and were vacuum distilled prior to use. Bovine Fn (Catalog Number: 1030Fn) was obtained from R&D Systems. Fn antibody (C6F10: sc-73611) was obtained from Santa Cruz. The water used in the experiment was purified by a Milli-Q water system (Millipore, USA). Quartz, glass and silicon wafers were cleaned in piranha solution (7:3 v/v% H_2_SO_4_/H_2_O_2_) (Caution! Piranha is a strong oxidizer and should not be stored in a closed container). After being copiously rinsed with water, they were dried under a smooth stream of N_2_.

### Synthesis of PHEMA brushes

The synthesis of PHEMA brushes was accomplished according to the method reported previously [20]. The thickness of PHEMA brushes was varied by changing the polymerization time according to the previous results [20].

### Film thickness

The thicknesses of the resulting polymer films were characterized by variable-angle spectroscopic ellipsometry (VASE, J.A. Woollam Inc., Lincoln, NE, USA) equipped with a focusing probe. Measurements were conducted under dry condition at three angles of incidence (65°, 70° and 75°) in the spectral range of 300–1700 nm. Spectroscopic scans were taken before (ATRP initiators-grafted surface) and after PHEMA polymerization. The measurements were fitted with the WVASE32 analysis software using a multilayer model for a silicon dioxide layer on silicon and a polymer adlayer. The PHEMA adlayer thickness was determined by a Cauchy model (*A*_n_ = 1.45, *B*_n_ = 0.01 and *C*_n_ = 0). PHEMA films of different thickness were measured and analyzed. Triplicate measurements were also carried out on each sample.

### Protein adsorption

The thickness of adsorbed proteins was measured by ellipsometry according to the method reported previously [1]. Briefly, the PHEMA-grafted slides were incubated in protein solutions (30 μg/ml Fn, 4 mg/ml BSA and 10% FBS) at 37 C for 12 h, respectively. After being washed with PBS to remove the non-adsorbed proteins, and water to remove the free ions, the slides were blow-dried with a nitrogen flow. The thickness of protein adlayer was obtained by VASE, by subtracting the thickness of the initial polymer adlayer (thickness of PHEMA) from the total thickness after exposure to the protein solution. The experiments were carried out in triplicate.

To quantify the adsorption amount of Fn on the PHEMA-grafted surface from 10% serum, the Fn molecules were labeled with ^125^I (ICN Pharmaceuticals, Irvine, CA, USA) using a method reported previously [22]. Free iodide was removed by using the AG 1-X4 column (Bio-Rad Labora-166 tories, Hercules, CA, USA). To study the Fn adsorption from serum, the labeled Fn molecules were mixed with unlabeled ones to give a total concentration of 30 μg/ml (the Fn in serum and R&D System are both from bovine and thereby are identical). The substrate was incubated in the protein solution at 37 C for 2 h, rinsed three times in PBS (10 min each), sucked with filter papers and then transferred to clean tubes for radioactivity measurement by WIZARD^2^ automatic γ-Counters (Perkin-Elmer 1480, USA).

### Activity of Fn detected by ELISA

The RGD activity in Fn adsorbed on the PHEMA films was evaluated by using the Fn antibody (C6F10, sc-73611) clones as the first antibody and the IgG-HRP as the second antibody (1:5000). Briefly, after the PHEMA brushes adsorbed with the FBS proteins were incubated in 1% BSA/PBS solution for 4 h, they were incubated in Fn antibody solution (diluted with 5% BSA/PBS with a ratio of 1:200) at 37°C for 1 h, followed by three washes with 1% BSA/PBS solution. The slides were then maintained in IgG-HRP solution (diluted with 1% BSA/PBS with a ratio of 1:5000) at 37°C for 40 min, and followed with five washes with PBS. Finally, the slides were immersed in 3,3′,5,5′-tetramethylbenzidine (TMB) solution at 37°C for 30 min, and followed by addition of the TMB stop solution (0.5 M H_2_SO_4_). The solution was then distributed in triplicate to a 96-well plate (200 μl), and the optical density (O.D) at 450 nm was measured by a Microplate reader (Biorad model 680). The experiments were repeated triplicate.

### Protein adhesion force measurement by AFM

The adhesion force between Fn and PHEMA films was measured by using atomic force microscopy (AFM) with a Fn-modified tip [23]. The commercially available silicon nitride triangular cantilever (200 μm long) used in this study had a pyramid-like-shaped tip, whose spring constant was 0.08 N/m. Briefly, the Fn molecules were covalently immobilized on the gold-coated and 11-mercapto-undecanoic acid modified Si_3_N_4_ tip via the reaction between the amino groups in Fn and the carboxyl groups on the tip. In detail, the oxygen plasma-treated cantilever was incubated in 1 mg/ml 11-mercapto-undecanoic acid in ethanol for 2 h, and washed with ethanol and water five times. After the generated carboxylic groups on the cantilever were activated in 1-ethyl-3-(3-dimethylaminopropyl) carbodiimide hydrochloride (0.1 mol/l) and *N*-hydroxysuccinimide (0.05 mol/l) solution for 1 h, the cantilever was immediately incubated in 30 μg/ml Fn/PBS solution at 37°C for 2 h.

The adhesion force between Fn and PHEMA films was measured in PBS (pH = 7.4, 10 mM) from the approach and retraction traces of the force-versus-distance (*f–d*) curve at room temperature. The adhesion force is defined as the shift value of deflection in the retraction trace of the *f–d* curves from the bottom of the retraction line. In each measurement, more than 100 of the *f–d* curves were collected to get the average value.

### Cell adhesion

Human VSMCs were obtained from the Cell Bank of Typical Culture Collection of Chinese Academy of Sciences (Shanghai, China). The cells were maintained with regular growth medium consisting of high-glucose Dulbecco’s modified eagle medium (Gibco, USA), supplemented with 10% fetal bovine serum (FBS, Sijiqing Inc., Hangzhou, China), 100 U/ml penicillin and 100 µg/ml streptomycin, and cultured at 37°C in a 5% CO_2_ humidified environment. Before cell culture, the PHEMA-grafted slides (1 × 1 cm^2^) were sterilized in a 24-well culture plate under the UV irradiation for 1 h. Then, the VSMCs were seeded into the wells at a density of 2.5 × 10^4^ cells/well and incubated at 37°C in a 5% CO_2_ humidified environment. The cell spreading area was obtained by analyzing the images of cells with fluorescein diacetate (FDA) dying.

### Cell migration

Cell migration was observed according to the methods reported previously [24, 25]. Briefly, the VSMCs were seeded at a density of 5 × 10^3^ cells/cm^2^. Approximately 12 h after the cell plating in 10% FBS with and without Fn-depleted mediums, the cell migration trajectories were *in situ* monitored using a time-lapse phase-contrast microscope (DMI 6000B, Leica) equipped with an incubation chamber (37°C and 5% CO_2_ humidified atmosphere) over a period of 12 h, respectively.

The VSMC trajectories were reconstructed from the center positions of individual cells through the whole observation time. The cell migration distance *S* was calculated by Image pro Plus software according to the following equation (Eq. 1) at 30 min time intervals over the observation time 12 h (*t* = 12).

(1) $$\text{S}=\overset{t}{\underset{i=1}{\sum }}\sqrt[{2}]{{\left(x_{i+1}-x_{i}\right)}^{2}+{\left(y_{i+1}-y_{i}\right)}^{2}}.$$

At least 60 cells were calculated for each sample. The cell migration rate (*ν*) was thus obtained by *ν* = *S/t* (μm/h).

#### Statistical analysis

The experimental data are expressed as mean ± standard deviation and the significant difference between groups was analyzed using one-way analysis of variance (ANOVA) (for two groups) and two-way ANOVA (for more than two groups) in the Origin software, and the statistical significance was set as *P* < 0.05.

## Results

### Preparation and characterization of PHEMA-grafted surfaces

The hydroxyl groups on the substrate were reacted with APTES for desired time, yielding -NH_2_ end-capped surface. The amine groups on the substrate were further reacted with BIBB, resulting in ATRP-initiators grafted surface. Their density was dependent on the change of the amino density before and after BIBB immobilization. The results from previous work showed that the BIBB density was 1.5/nm^2^ [20]. The thickness of the PHEMA brushes increases linearly along with the prolongation of polymerization time, and thereby the PHEMA surface with different thickness was easily fabricated by controlling the time [20]. The water contact angle on the PHEMA surface decreased with increase of the PHEMA thickness and reached a plateau ( ∼50°) at 20 nm [20]. As a hydrophilic polymer, the PHEMA film can swell two times in water compared with its initial thickness in a dry state.

The grafting density was estimated according to the previous work and an assumed chain initiation efficiency of approximately 10% [26], the density of PHEMA brush (*σ*) was nearly 0.15/nm^2^. The number-average molecular weight (*M*_n_) was estimated from Equation (2) [26].

(2) $$M_{\text{n}}=\frac{h\rho N_{\text{A}}}{\sigma },$$

where *h* is the dry thickness of grafting layer, *ρ* is the bulk density of PHEMA (1.07 g/cm^3^), *N*_A_ is the Avogadro number, *M*_n_ is the number-average molecular weight and σ is the density of PHEMA brush layer.

The results are shown in Table 1. The estimated *M*_n_ of 3, 6, 15 and 20 nm PHEMA is 12833, 25765, 64414 and 85885 Da, respectively. Moreover, the PHEMA density can be further converted into the distance between two adjacent PHEMA chains (*L*) according to Equation (3):

(3) $$L={\left(\frac{\text{2}}{\sqrt{\text{3}}\sigma_{}}\right)}^{\text{0.5}}.$$

It is known that the conformation of grafted PHEMA molecules is determined by *L*/2*R*_g_ (*L*/*2R*_g_ > 1, the adjacent polymers in the hydrated state do not overlap and can rotate freely without disturbance, forming a mushroom-like regime; *L*/*2R*_g_ < 1, the polymers are in a crowded state, forming a so-called ‘brush’ regime). *R*_g_ is the radius of gyration of PHEMA chains. It is calculated from the known relation between *R*_g_ and molecular weight of PHEMA, which is similar to the PEG [27].

(4) $$R_{\text{g}}=0.181X_{n}{}^{0.5},$$

where *X_n_* is the degree of polymerization.

**Table 1.** physiochemical properties of surface-grafted PHEMA brushes with different thickness

Dry thickness *h* (nm)	Estimated molecular weight *M*_n_ [g/mol]	Radius of hydration *R*_g_ (nm)	Distance *L* (nm)	*L*/2*R*_g_	Configuration
0	NA	NA	NA	NA	NA
3	12 833	1.8	2.8	0.78	Brush
6	25 765	2.5	2.8	0.56	Brush
15	64 414	4.0	2.8	0.35	Brush
20	85 885	4.6	2.8	0.30	Brush

The estimated *R*_g_ of 3, 6, 15 and 20 nm PHEMA films is 1.8, 2.5, 4.0 and 4.6 nm. Therefore, the *L/2R*_g_ is 0.78, 0.56, 0.35 and 0.3, respectively, revealing that all the PHEMA molecules are in a crowded state, and thereby their rotation shall be interfered with each other, enabling the PHEMA chains to stretch out and forming a so-called ‘brush’ regime (Table 1).

### Protein adsorption

**Figure 1.**
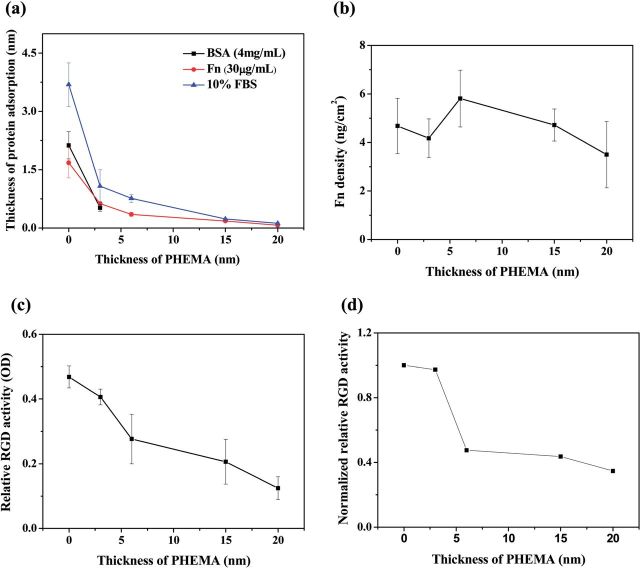
(a) Thickness of proteins adsorbed on PHEMA brushes with different thicknesses. (b) Density of Fn adsorbed from 10% FBS on PHEMA brushes detected by RIA. (c) The relative RGD activity (OD) of Fn adsorbed from 10% FBS on PHEMA brushes. (d) The normalized relative RGD activity of adsorbed Fn to its amount (OD in (c)/adsorption amount in (b)).

The thickness of proteins adsorbed on PHEMA brushes of different thickness was detected by ellipsometry. Figure 1a shows that the amount of proteins (Fn and 10% FBS) adsorbed on the PHEM bushes decreased with increase of the thickness of PHEMA. The amounts of BSA adsorbed on the bare substrate (0 nm) and 3 nm PHEMA brushes were 2.13 ± 0.35 and 0.52 ± 0.09 nm, respectively. No BSA could be detected by ellipsometry when the thickness of PHEMA was 6 nm or larger.

To quantify the Fn adsorption from 10% FBS on the PHEMA brushes, the radio immunoassay (RIA) was used (Fig. 1b). The Fn showed a different adsorption pattern from the 10% FBS (Fig. 1b) as from the pure Fn solution (30 μg/ml, Fig. 1a). The amount of Fn decreased initially with the increase of PHEMA thickness. However, it increased again on the 6-nm thick PHEMA brushes, the highest amount compared with all other PHEMA brushes (Fig. 1b).

It is known that the function of adsorbed Fn molecules is not only influenced by their amount, but also the remained bioactivity. Hence, the ELISA method was used to further evaluate the relative RGD activity in the adsorbed Fn (Fig. 1c). The relative RGD activity in the Fn adsorbed from 10% FBS on the PHEMA brushes decreased monotonously along with the increase of PHEMA thickness. By normalization of the relative RGD activity to the adsorbed Fn amounts, the normalized relative RGD activity is further compared in Fig. 1d. The Fn adsorbed on the thinner PHEMA brushes (0, 3 nm) had far larger relative RGD activity (*P* < 0.05) than those adsorbed on the thicker ones (≥6 nm), and there was a sharp transition above 6 nm of PHEMA brushes.

### Fn adhesion force

**Figure 2.**
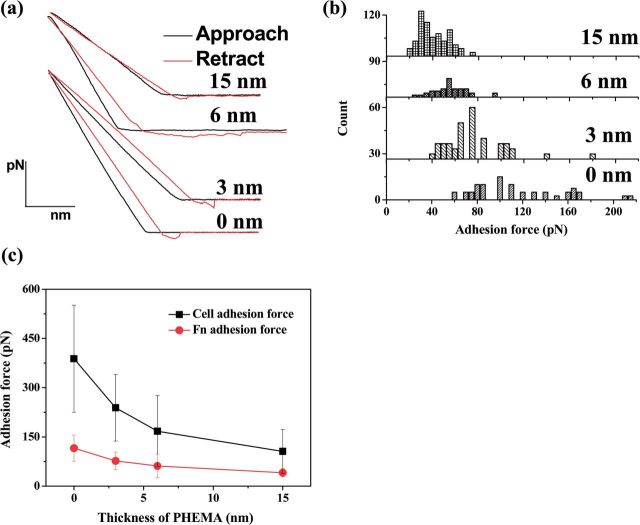
(a) Representative force-versus-distance curves between Fn-immobilized tip and PHEMA brushes with different thicknesses. (b) Histograms of adhesion force of Fn on PHEMA brushes. (c) Relationship between adhesion force of Fn and cell adhesion (data from literature [20]; the experimental details can be found thereof too) on different thickness of PHEMA brushes. The total number of Fn modified on the AFM tips remains same for all the measurement because of the same treatment of these tips.

Protein–surface interactions are fundamental to numerous biological processes and disease pathologies [28]. More specifically, the cell protein interaction may be governed by the conformational nature of adsorbed proteins, which is directed by the properties of the underlying properties of surface [29]. Therefore, it is important to measure the adhesion force between the surface of biomaterials and proteins. Previous work showed that the cell adhesion force is closely associated with the adsorbed Fn molecules [30]. To explore the relationship, the adhesion force of Fn on the PHEMA brushes of different thickness was determined by AFM. Figure 2a shows the representative force–distance curves for the approach and retraction of the Fn-immobilized tip from the PHEMA brushes. The adhesion force was defined by the maximum pull-off force in the force–distance curve during the retraction of the Fn-immobilized tip, whose distribution is shown in Fig. 2b and values are averaged and shown in Fig. 2c. The average adhesion force of Fn decreased monotonously along with the increase of thickness of PHEMA brushes, and changed from 115.5 pN (the bare substrate) to 40.9 pN (20 nm PHEMA brushes). Figure 2c further compares the Fn adhesion force with the cell adhesion force, revealing that they have the similar alteration tendency on the PHEMA brushes although the cell adhesion force is much larger (388 pN on 0 nm PHEMA brush surface) due to the different methods used. This result shows that the adhesion strength of cells is greatly contributed by the adhesion strength of the previously adsorbed Fn, which is known to interact with cell surface via the RGD domains.

### *In vitro* cell culture

**Figure 3.**
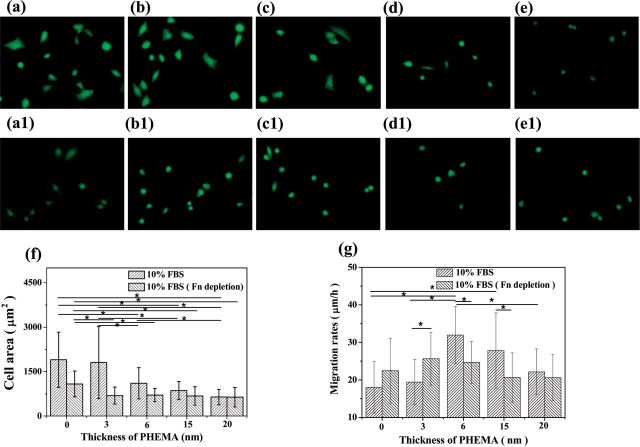
Images of VSMCs adhered on PHEMA brushes with thicknesses of 0, 3, 6, 15, 20 nm in (a–e) 10% FBS medium and (a1–e1) 10% Fn-depleted FBS medium, respectively. (f) Spreading area and (g) migration rate of VSMCs on PHEMA brushes with normal culture medium (10% FBS) and Fn-depleted medium, respectively. Asterisk indicates significant difference at *P* < 0.05.

VSMCs are essential for blood vessels for vessel resetting in physiological conditions such as pregnancy and exercise, or after vascular injury [31]. For some vascular surgeries, VSMCs migration may induce thrombosis and intimal hyperplasia, especially revascularization for small diameter blood vessel tissue engineering [32]. So investigation of the VSMCs behaviors and understanding of the underlying mechanism are very important and meaningful. In order to evaluate whether the adsorbed Fn plays a significant role on mediating the cell behaviors on the PHEMA brushes, VSMCs were cultured on the PHEMA brushes in normal 10% FBS and Fn-depleted 10% FBS for 24 h, respectively. The fluorescent images of VSMCs were taken after FDA staining (Fig. 3a–e, a1–e1). Compared with the more spreading cell morphology on the 0 and 3 nm PHEMA brushes, most of the VSMCs adhered on the 6, 15 and 20 nm PHEMA brushes presented round shape and less spreading (Fig. 3a–e), especially on the 20 nm PHEMA brushes (Fig. 3e). In the Fn-depleted medium, most of the VSMCs showed round shape and spread badly regardless of the thickness of PHEMA brushes, except on the bare substrate. The statistical results of spreading area of VSMCs are presented in Fig. 3f. The cell spreading area on the 0 and 3 nm PHEMA brushes was significantly larger than those on the 15 and 20 nm PHEMA brushes in the normal culture medium (*P* < 0.05), and that on the 6 nm PHEMA brushes showed a moderate value. In the Fn-depleted medium, the cell spreading area on the 0, 3 and 6 nm PHEMA brushes was significantly decreased (*P* < 0.05), and showed no significant difference (*P* > 0.05) among all the 3, 6, 15 and 20 nm PHEMA brushes. Moreover, the cell spreading area on the bare substrate without PHEMA grafting was still significantly larger (*P* < 0.05) than those on the PHEMA brushes. This phenomenon may be resulted from the adsorption of other types of adhesive proteins such as laminin and vitronectin, which can be resisted on the PHEMA brushes as well.

Figure 3g shows that the cells migration rate increased initially and then decreased along with the increase of thickness of PHEMA brushes regardless of the Fn depletion. However, the detail alteration pattern is different. The VSMCs migrated fastest and slowest on the 6 nm PHEMA brushes and 0 nm bare substrate in the normal medium, respectively, which is consistent with previous observation [20]. In the Fn-depleted medium, the cell migration rate on the 6 and 15 nm PHEMA brushes significantly decreased (*P* < 0.05), kept unchanged on the 20 nm PHEMA brushes (*P* > 0.05), whereas significantly enhanced on the 0 and 3 nm PHEMA brushes (*P* < 0.05).

## Discussion

It is very convenient to control the thickness of PHEMA brushes by controlling the polymerization time of ATRP [20]. The grafting density of PHEMA brushes (σ) was nearly 0.15/nm^2^ estimated from the BIBB density. The estimated R_g_ of 3, 6, 15 and 20 nm PHEMA brushes was 1.8, 2.5, 4.0 and 4.6 nm, and L/2R_g_ was 0.78, 0.56, 0.35 and 0.3, respectively. Therefore, all the PHEMA molecules with the thicknesses of 3, 6, 15 and 20 nm are in a crowded state, and thereby their rotation shall be interfered with each other, enabling the PHEMA chains to stretch out and forming a so-called ‘brush’ regime (Table 1). In this case, the exposed substrate area is effectively zero.

BSA is the most abundant serum proteins, which is commonly modeled as an ellipsoid of a dimension of 14 × 4 × 4 nm^3^ [33] and is also among the most significant proteins that adsorb on biomaterial surfaces. Fn includes two almost identical subunits of about 220 kDa each, and is crosslinked by disulfide bonds in the C-terminal with a maximum length of 140 nm and average diameter of the strand 2 nm for each subunit [34, 35]. Each subunit of Fn contains several cell binding regions such as RGD domains [29, 36, 37], to which cells can attach directly through integrins α_5_β_1_ or α_v_β_3_ [38, 39]. It also has numerous functions involving in cell growth, migration and differentiation. The amount of proteins adsorbed from single protein solution and 10% FBS on the PHEMA brushes was measured by VASE which gives an absolute value of total protein adlayer thickness, irrespective of the conformational change of proteins upon adsorption. The results from Fig. 1a show that the amount of adsorbed proteins (Fn and 10% FBS) gradually decreased as the thickness of PHEMA increasing. However, adsorption of BSA on 6, 15 and 20-nm thick PHEMA brushes was not detectable, conveying that it is too large to penetrate the thick PHEMA brushes and adsorb onto the substrate. However, Fn could still adsorb due to its flexible and special structure to adopt different conformations depending on the environmental conditions and different properties of biomaterials surface [27]. In this study, Fn should be able to change its conformation and unfold the motif in order to adsorb maximally on the thicker PHEMA brushes. The amount of Fn adsorbed on the 6-nm thick PHEMA brushes was the highest from 10% FBS detected by RIA, which is different to that in the pure Fn solution. This fact reflects the competitive adsorption between Fn and other types of proteins in the serum. These results show that even all the PHEMA molecules are in a crowded state and no bare substrate should be exposed directly, the protein molecules are still able to adsorb due to the ternary and secondary adsorption. These results are in common with previous studies, in which PHEMA film of high thickness can effectively resist nonspecific bacterial adhesion and protein adsorption from single proteins such as aprotinin, but they lose their excellent antifouling performance in undiluted human blood serum and plasma [15, 40].

To correlate the function of adsorbed Fn on the cell responses, VSMCs were cultured on the PHEMA brushes with normal FBS-containing medium and Fn-depleted FBS medium to compare the difference in cell adhesion, spreading and migration. The VSMCs spreading area significantly decreased in the Fn-depleted serum regardless of the thickness of PHEMA brushes (Fig. 3f). However, the cell migration rate was significantly enhanced on the 0 and 3 nm PHEMA brushes, whereas decreased on the 6 and 15 nm PHEMA brushes, and was kept unchanged on the 20 nm PHEMA brushes (Fig. 3g). These results reveal that Fn with the ease of RGD exposure could basically enhance the cell adhesion and spreading, but would slow down the cell mobility as shown on the 0 and 3 nm PHEMA brushes. The Fn adhesion force and the cell adhesion force had a similar alteration tendency on the PHEMA brushes, revealing that the adhesion strength of cells is greatly contributed by the adhesion strength of the previously adsorbed Fn, which influences the cell migration.

**Figure 4.**
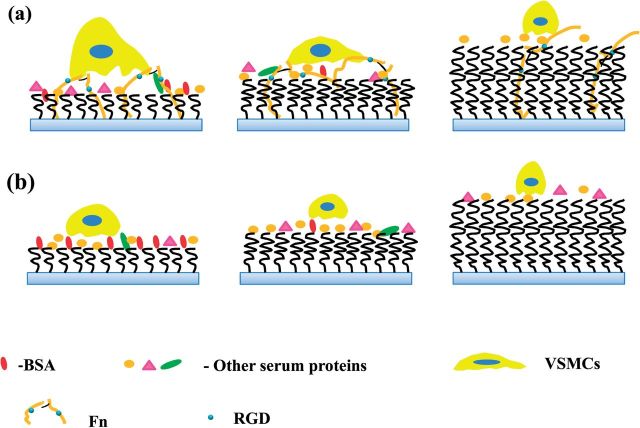
Schematic diagrams of VSMCs being cultured on the PHEMA brushes with different thicknesses in (a) normal medium and (b) Fn-depleted medium.

All these data strongly suggest that the difference in Fn adsorption should take the major role on the different adhesion and migration of VSMCs on the PHEMA brushes. It is known that mediation of cell migration and outgrowth by the adsorbed Fn on materials is mainly implemented via the RGD domains in the Fn molecule, which interact with the receptors in cells [41]. Although the amount of Fn adsorbed from FBS-containing medium (Fig. 1a) on the PHEMA brushes of different thickness had no significantly difference from 10% FBS, the relative RGD activity of the adsorbed Fn decreased with the increase of PHEMA thickness. The Fn molecule has to change its conformation largely to adsorb on the thicker PHEMA brushes, and thereby many of the RGD domains are buried inside the polymer brushes, leading to the poorer cell adhesion and spreading. The Fn molecules adsorbed on the top surface of bare substrate or the thinner PHEMA brushes (3 nm) will be easy to expose their RGD domains and thereby have high relative RGD activity, resulting in stronger cell adhesion and good cell spreading (Fig. 4 Left). This stronger interaction between the VSMCs and thinner PHEMA brushes bridged by the adhesive Fn, at least partially, however, will lead to the difficulty in release of the rear end of cells during migration, and thereby the slower cell mobility (Fig. 4 Left). The adsorbed Fn molecules on the 6 nm PHEMA brushes can bury some RGD domains and have the moderate RGD activity, and provide the moderate (and appropriate) adhesion force, enabling the cells to migrate fastest (Fig. 4 Middle). The adsorbed Fn molecules on thicker PHEMA brushes (15 and 20 nm) will bury more RGD domains and have the lowest RGD activity, and will not be able to provide strong enough adhesion force for the cell mobility. Therefore, the difference in Fn adsorption on the different PHEMA brushes plays a critical role on mediating the cell adhesion, spreading and migration, although other types of adhesive proteins may also contribute to some extent.

## Conclusion

A quantitative investigation of Fn adsorption and cell adhesion and migration on PHEMA brushes has been carried out in this study. The grafting density of PHEMA brushes (σ) was nearly 0.15/nm^2^, and the 3, 6, 15 and 20 nm PHEMA were all in a brush state. The amounts of adsorbed Fn and FBS decreased monotonously along with the increase of thickness of PHEMA brushes, whereas adsorption of BSA was not detectable when the thickness of PHEMA brushes was larger than 6 nm by ellipsometry. Although on the 6 nm PHEMA brushes the adsorption of Fn from 10% FBS was the highest, no significant difference in Fn adsorption amount was found by RIA. Combined with ELISA detection, the normalized relative RGD activity decreased monotonously along with the increase of PHEMA thickness. When the adhesion force of VSMCs, which is mediated by the adsorbed Fn and its relative activity, was appropriate such as on the 6 nm PHEMA brushes, the smooth muscle cells had the highest mobility. By contrast, the cell mobility was quite low on either the 0 and 3 nm PHEMA brushes with high RGD activity, which causes too strong cell adhesion and the difficulty of rear end release of the cells, or the 20 nm PHEMA brushes which are not able to exert strong enough adhesion force for cell migration. All the results suggest that the adsorption manner of Fn, its conformation and activity contribute significantly to the responses of VSMCs to the PHEMA brushes of different thickness in terms of adhesion, spreading and migration.
